# Confrontation of fibroblasts with cancer cells in vitro: gene network analysis of transcriptome changes and differential capacity to inhibit tumor growth

**DOI:** 10.1186/s13046-015-0178-x

**Published:** 2015-06-18

**Authors:** Andrey Alexeyenko, Twana Alkasalias, Tatiana Pavlova, Laszlo Szekely, Vladimir Kashuba, Helene Rundqvist, Peter Wiklund, Lars Egevad, Peter Csermely, Tamas Korcsmaros, Hayrettin Guven, George Klein

**Affiliations:** Department of Microbiology, Tumor and Cell Biology (MTC), Karolinska Institutet, Stockholm, Sweden; Bioinformatics Infrastructure for Life Sciences, Science for Life Laboratory, Karolinska Institutet, Box 1031, 171 21 Solna, Sweden; College of Science, Department of Biology, Salahaddin University, Erbil, Kurdistan-Iraq; Institute of Molecular Biology and Genetics, UNAS, Kiev, Ukraine; Department of Cell and Molecular Biology (CMB), Karolinska Institutet, Stockholm, Sweden; Department of Molecular Medicine and Surgery, section of Urology, Karolinska Institutet, Stockholm, Sweden; Department of Oncology-Pathology, Karolinska Institutet, Stockholm, Sweden; Department of Medical Chemistry, Semmelweis University, P.O. Box 260, H-1444 Budapest 8, Hungary; TGAC, The Genome Analysis Centre, Norwich Research Park, Norwich, UK; Gut Health and Food Safety Programme, Institute of Food Research, Norwich Research Park, Norwich, UK

**Keywords:** Fibroblast, Stroma, Transcriptome, Systems biology, Differential expression, Cancer associated fibroblasts (CAFs), Cancer cell growth

## Abstract

**Background:**

There is growing evidence that emerging malignancies in solid tissues might be kept under control by physical intercellular contacts with normal fibroblasts.

**Methods:**

Here we characterize transcriptional landscapes of fibroblasts that confronted cancer cells. We studied four pairs of *in vitro* and *ex vivo* fibroblast lines which, within each pair, differed in their capacity to inhibit cancer cells. The natural process was modeled in vitro by confronting the fibroblasts with PC-3 cancer cells. Fibroblast transcriptomes were recorded by Affymetrix microarrays and then investigated using network analysis.

**Results:**

The network enrichment analysis allowed us to separate confrontation- and inhibition-specific components of the fibroblast transcriptional response. Confrontation-specific differences were stronger and were characterized by changes in a number of pathways, including Rho, the YAP/TAZ cascade, NF-kB, and TGF-beta signaling, as well as the transcription factor RELA. Inhibition-specific differences were more subtle and characterized by involvement of Rho signaling at the pathway level and by potential individual regulators such as IL6, MAPK8, MAP2K4, PRKCA, JUN, STAT3, and STAT5A.

**Conclusions:**

We investigated the interaction between cancer cells and fibroblasts in order to shed light on the potential mechanisms and explain the differential inhibitory capacity of the latter, which enabled both a holistic view on the process and details at the gene/protein level. The combination of our methods pointed to proteins, such as members of the Rho pathway, pro-inflammatory signature and the YAP1/TAZ cascade, that warrant further investigation via tools of experimental perturbation. We also demonstrated functional congruence between the in vitro and ex vivo models.

The microarray data are made available via the Gene Expression Omnibus as GSE57199.

**Electronic supplementary material:**

The online version of this article (doi:10.1186/s13046-015-0178-x) contains supplementary material, which is available to authorized users.

## Background

Stoker et al. have shown that normal fibroblasts can inhibit the growth of cancer cells in vitro [1] and termed this effect as neighbor suppression. Recently, our group has confirmed this finding in a high-throughput system [2, 3] and showed that fibroblasts from puncture biopsies, taken from patients with prostatic cancer, had less inhibitory effect than normal skin fibroblasts. In normal tissues, the inhibitory effect differed depending on the original location of the fibroblasts. Hernia fibroblasts were less inhibitory than skin fibroblasts. Flaberg et al. [3] also showed that fibroblasts differing in their ability to inhibit tumor growth and motility can be isolated from the telomerase immortalized foreskin fibroblast line, BJ/TERT on the basis of their morphology. The more inhibitory sublines were designated as whirly (Wh), the less inhibitory as crossy (Cr). In this paper these two lines will be referred to as “*in vitro*” cells, whereas 6 other lines derived from recent biopsies will be referred to as “*ex vivo*“cells.

It is known that the normal tumor inhibitory microenvironment becomes “corrupted” during tumor development. This is reflected by appearance of a large and heterogeneous category of cancer-associated fibroblasts (CAFs) that fail to inhibit tumor growth or actually stimulate it. The molecular basis of this functional difference is not known. CAFs were shown to express markers associated with wound healing [4], inflammation [5], and epithelial-mesenchymal transition [6]. In an *in situ* analysis of antibody-stained tumor images from the Human Protein Atlas we have identified 12 new CAF markers expressed in cancer stroma but not in normal fibroblasts [7]. In the most recent work we studied protein factors that might be closely responsible for the cancer cell-fibroblast interaction and could distinguish between extracellular matrix based and soluble ones [8].

In order to examine the role of major genes and pathways that shape the CAF-tumor interaction and influence the tumor inhibitory capacity of fibroblasts, the 2 *in vitro* and 6 *ex vivo* fibroblasts were co-cultivated with a prostate cancer cell line *in vitro*. Transcriptome profiling of fibroblast cells was performed with Affymetrix microarrays.

The present study aimed at:

i. Characterizing the transcriptome response of the eight fibroblast lines to tumor cells in the course of *in vitro* co-culturing “confrontation experiment”;
ii. Determining the transcriptional correlates of differential inhibition capacity;
iii. Examining the prognostic and, potentially, treatment-relevant significance of the genes highlighted by the steps (i) and (ii) above, by utilizing public resources of clinical and molecular (gene expression) data from The Cancer Genome Atlas [9].

Global analysis of transcription usually generates long lists of differentially expressed (DEG) genes. Their common features can be revealed by gene set enrichment analysis (GSEA) against functionally annotated gene sets, such as Gene Ontology terms [10] or KEGG pathways [11] that significantly overlap with lists of DEGs are then used to characterize the latter. Known drawbacks of GSEA are that 1) most of the genes do not have specific annotations in the databases, 2) the overlap can only be observed for genes that differ transcriptionally in the relevant comparisons, which omits proteins that function via other mechanisms, e.g. by phosphorylation, and 3) the statistical power of the analysis is limited by the sizes of functional gene sets (FGS). The smaller a gene set, the harder is it to prove its significance in GSEA – whereas a deeper study would usually focus on compact pathways. As an example from Reactome database [12], the “mitotic cell cycle” pathway consisted of 329 genes, whereas only 121 and 43 of these genes constituted “cell cycle checkpoints” and “G2-M checkpoint”, respectively. The latter two are much more difficult to identify in GSEA.

In order to overcome these limitations, we recently extended GSEA to network enrichment analysis (NEA) [13]. The key difference is that GSEA calculates the significance of overlap of member genes between DEGS and a functional gene set, whereas the significance in NEA is evaluated by functional connections (network links) that have been identified between genes of the two groups. The source of functional connections for NEA is a global network of functional coupling between genes and proteins, such as FunCoup [14, 15].

This generalization allows NEA to circumvent the above mentioned drawbacks of GSEA by considering nearly all known genes and proteins and their molecular *modi operandi*. As demonstrated in [13], the statistical power of NEA is an order of magnitude higher compared to GSEA. In addition, the method can analyze individual DEGs and key functional regulators against gene sets. These features were indispensable in the context of the present work, since we were looking for fine-grained mechanisms shaped by a few DEGs and small pathways. In particular, when the list of binding sites predicted for transcription factors was presented as a set of network edges, the NEA methodology permitted the identification of binding sites enriched in lists of DEGs. NEA can also demonstrate that different DEGs, when combined in a top-ranking list, relate to each other. Special statistical tests rejected the null hypothesis that the list members merely represented random sets of genes, which thus indicated their functional coherence (i.e. appearance as a network module).

In this article, we investigated the expression of genes in fibroblasts that differed in their cancer inhibitory capacity. Observations were made before and after fibroblast co-cultivation (confrontation) with tumor cells. Then the fibroblast transcriptomes were analyzed by NEA, both for their individual and collective properties. We also tested the prognostic significance of the identified key genes in ten different cancers. Our overall aim was to generate biological hypotheses for further experimental testing and to suggest genes that would warrant further investigation.

## Methods

### Primary human fibroblasts

Primary human fibroblasts were cultivated from diagnostic biopsy samples or from surgically removed tissue pieces from different anatomic locations of pediatric and adult patients. Primary skin fibroblasts were isolated from a pediatric patient wound site skin (dubbed as pediatric skin fibroblast PdSFB). From the same pediatric patient, fibroblasts were also obtained by resection during umbilical hernia surgery (pediatric hernia fibroblast PdHFB). Fibroblasts HS68, derived from skin of a healthy donor (available in ATCC), were provided by Prof. Kenneth Wu (NHRI, Taiwan).

Fibroblasts PrNFB1 (prostate normal fibroblast 1), PrTFB1 (prostate tumor fibroblast 1) and PrTFB2 (prostate tumor fibroblast 2) were derived from the following prostate biopsies:

- PrNFB1 from a non-tumor (normal) prostate area in a patient with prostate carcinoma,
- PrTFB1 from a tumor area of the same patient, and
- PrTFB2 from a tumor prostate area in another patient with prostate carcinoma.

These primary human prostate fibroblast cultures were established using the method by Tuxhorn et al. [16] as previously described. In brief, fresh prostate tissue derived from radical prostatectomy was diced in small pieces of about one mm^3^. These pieces were put into 6-well tissue culture plates and were fixed in the well under a cover slide. Then 1.5 ml Bfs medium [DMEM (Hyclone) supplemented with 5 % FBS (Hyclone), 5 % Nu Serum (BD Biosciences), 5 mg/ml Insulin, 0.5 μg/ml Testosterone, 4 mM L-glutamine and 1× Penicillin/ Streptomycin (all from Sigma)] was added to each well, and the tissue pieces were incubated at 37 °C with 5 % CO_2_. Fibroblast-like cells started to migrate out from the tissue between 5 and 15 days and were passaged when confluent. The fibroblast nature of the tissue-derived cell cultures was verified by their fibroblast- characteristic morphology and the expression of fibroblast- markers such as PDGFR-beta, alpha-SMA, but not e.g. e-cadherin [8].

After generation of primary fibroblasts, they were cultured in IMDM supplemented with 20 % FBS and antibiotics. Primary fibroblasts of passage number below eleven were used in the tumor/fibroblast co-culture assays. All primary fibroblasts were additionally transduced with GFP (the green fluorescent protein) using third generation lentivirus produced in our facilities. The lentivirus vectors were generous gift from Prof. Galina Selivanova (Karolinska Institute).

### Ethical permissions

The primary fibroblasts were obtained from surgical or diagnostic rest material. Informed consent from either patients or parents was received. All samples were coded and only information about age, gender and site of origin were given to investigators. The handling of these materials was approved both by the Regional and Institutional Ethics Committee, University of Debrecen Medical and Health Science Center (DEOEC RKEB/IKEB no. 2918–2009) (for the PdSFB and PdHFB fibroblasts) and the Regional Ethical Review Board in Stockholm (No. 2010–087).

### Isolation of subclones of BjhTERT fibroblasts

The maintenance of the human fibroblasts that were immortalized with the catalytic subunit of human telomerase (hTERT), BjhTERT, and the isolation of their subclones have been described previously [3]. Namely, we obtained the sub-clones by plating 1000 BjhTERT cells in a 10-cm tissue-culture dish in 10 ml Iscove’s modified Dulbecco’s medium containing 10 % fetal bovine serum, 100 μg/ml of penicillin/streptomycin. A few colonies started to appear after 2 to 3 weeks. After 4 weeks, the colonies were examined under light microscopy, and 48 colonies were removed from the dish using a pipette tip, and were cultured further. Out of these 48 subclones, we selected two that showed the ‘whirly’ (Wh1) and ‘crossy’ (Cr9) phenotypes. These whirly and crossy fibroblasts then maintained their phenotypic differences during serial passages.

### Co-culture inhibition assay

Tumor cell proliferation on fibroblast monolayers was analyzed in 384-well plates. Fibroblasts were plated in 80 μl complete medium and cultured for 5–6 days to form confluent and aged monolayers. H2AmRFP labeled PC-3 tumor cells were plated in fresh 80 μl complete medium on top of the fibroblast monolayers. The control wells contained 200 labeled tumor cells without fibroblasts. The inhibition score was calculated, as explained in details in [8], by dividing the number of tumor cells on day 5 with the number of tumor cells on day 0.

### Automated microscopy

Every well of the 384-well plate was imaged using a modified version of the automated microscope system. We used the Openlab automation Platefocus 10, developed by us. Images at 2.5× magnification (NA 0.08), covering the entire bottom-area of a well, were captured after seeding of tumor cells (day 0) and after 5 days of co-incubation with fibroblasts. At each time-point both transmitted light and fluorescence images were captured (excitation at 560 nm and emission at 600–620 nm for mRFP labeled cancer cells). The microscope platform was built using a Nikon microscope (Nikon, Tokyo, Japan), a programmable XY-table (Märzhauser, Germany) and a Retiga-4000RV (QImaging, Surrey, BC, Canada). Using binning 2, raw images were 1024 × 1024 pixels in size. The system was run on a Mac OS X, version 10.5.6, processor 2 × 3 GHz Quad-Core Intel Xeon.

### Affymetrix microarrays

Co-cultures were started by plating 0,5 × 10^6^ fibroblasts in collagen coated 10 cm dishes for 5–7 days. When cells reached full-confluency and the monolayers were matured, cells were washed 3× with PBS and 0,5 × 10^6^ PC-3 mRFP tumor cells were added for 4 days in complete medium. After 4 days of co-culture, the cells were washed 3× with PBS, trypsinized, and then the fibroblasts were sorted using BD FACSVantage (CA, USA). Total RNA was purified from flow cytometry sorted fibroblasts with and without PC-3 mRFP confrontation using the RNA Purification Kit (Ambion, USA) according to the manufacturer’s instructions. The Affymetrix Gene Chip WT Sense Target Labeling and Control Reagents kit (P/N 900652) was used for preparation of cDNA from 150 ng of total RNA. Array hybridization, washing, staining and scanning were performed on the Gene Titan system using the Gene Chip Human Gene 1.1 ST Array plate. Combining the probes, normalization and background correction were performed in Affymetrix Expression Console (v. 1.3.1) using the RMA method.

The data are available at Gene Expression Omnibus under Accession number GSE57199: https://www.ncbi.nlm.nih.gov/geo/query/acc.cgi?acc=GSE57199.

### Differential expression analysis

We detected differential expression between conditions of interest with R package OCplus [17]. This program enables fast calculation of both paired (which was applied when relevant) and independent t-tests across individual genes. Log_2_-tranformed values of the microarray dataset were normally distributed. We considered only genes with assigned HUGO symbol (by Affymetrix probe annotation). After visual investigation of the mean vs. variance plot, we detected a larger cluster of genes that stood out by low variance and low mean expression value across all the 16 samples. According to this observation, around 9000 genes with variance <0.1 and mean <4 were removed as likely “absent”. After this filtering, any correlation between mean and variance disappeared, which constituted a requirement for the *t*-test. The t-statistics were transformed to p-values and then adjusted for multiple testing using the procedure by Benjamini and Hochberg [18].

### Functional gene sets (FGS)

A larger compendium of pathways, such as KEGG, Reactome, BioCarta, WikiPathways, and otherwise defined functional sets (e.g. gene signatures) was downloaded from http://www.broadinstitute.org/gsea/msigdb/index.jsp as of April, 2011.

It was complemented with GO terms not larger than 500 genes, and custom gene sets compiled by us from literature. As the total size of this collection was more than 3000 sets, we then filtered it down to 82 pathways, which seemed to be the most relevant to this study.

### Global interaction network

The FunCoup network [14] integrated evidence of functional links using public data from multiple sources, high-throughput gene and protein expression profiling, sequence analysis (promoter and miRNA binding) and experimental evidence and annotations (physical contacts between proteins, sub-cellular co-localization etc.). We previously published results of a network benchmark [15] where we tested different combinations of known and predicted global networks by their ability to recover known members of functional gene sets. Here we use the best network version which was obtained by merging FunCoup edges at confidence higher than 0.5 with all known edges from the curated databases KEGG [11], PhosphoSite [19], CORUM [20], MSigDB [21], and HTRIdb [22]. The resulting union network had 974,427 functional links between 19,031 distinct HUGO gene symbols. Since these information sources could be either gene-, transcript-, or protein-specific and cover a wide range of cellular mechanisms and data mining approaches, the edges in the network represent unspecified “functional couplings” between nodes which could be both genes and proteins (referred to via HUGO gene symbols).

### Network enrichment analysis

We probabilistically estimated putative functional relations between pre-specified gene sets with the network enrichment analysis (NEA), a method presented earlier by Alexeyenko et al. [11, 22].

The standard z-score for the biological network connectivity between genes of a novel list (altered gene set, AGS) and genes of a known functional gene set (FGS, most commonly a pathway or a Gene Ontology term) could be quantified as a total number of links (edges) known in the global interaction network that connect any genes of AGS to any genes of FGS [13]. In the current work, we used a formula to calculate the number of links between AGS and FGS expected by chance:

$$ {x}^2=\frac{{\left[\left(n\right.\right]}_{\mathrm{AGS}\hbox{-} \mathrm{F}\mathrm{G}\mathrm{S}}-\widehat{\mathrm{n}}{\left.{}_{\mathrm{AGS}\hbox{-} \mathrm{F}\mathrm{G}\mathrm{S}}\right)}^2}{{\widehat{\mathrm{n}}}_{\mathrm{AGS}\hbox{-} \mathrm{F}\mathrm{G}\mathrm{S}}}+\frac{{\left[\left(!n\right.\right]}_{\mathrm{AGS}\hbox{-} \mathrm{F}\mathrm{G}\mathrm{S}}-!\widehat{\mathrm{n}}{\left.{}_{\mathrm{AGS}\hbox{-} \mathrm{F}\mathrm{G}\mathrm{S}}\right)}^2}{!{\widehat{\mathrm{n}}}_{\mathrm{AGS}\hbox{-} \mathrm{F}\mathrm{G}\mathrm{S}}}, $$

where *n*_*AGS-FGS*_ is the actual number of links between any genes of AGS and any genes of FGS, the respective number of links expected by chance is ${\widehat{\mathrm{n}}}_{\mathrm{AGS}\hbox{-} \mathrm{F}\mathrm{G}\mathrm{S}}=\frac{{\mathrm{N}}_{\mathrm{AGS}}*{\mathrm{N}}_{\mathrm{FGS}}}{2*{\mathrm{N}}_{\mathrm{total}}}$, and !*n* denotes “other than *n*”.

*N*_*AGS*_ and *N*_*FGS*_ report the sums of connectivities of individual nodes (genes) in AGS and FGS, respectively, while *N*_*total*_ is the number of edges in the whole network.

The statistic used cumulative connectivity values (total number of network links of all genes in the whole network) and was unbiased even if AGS and/or FGS are small and/or the network is sparse. Deviation of the actual value *n*_*AGS-FGS*_ from the observed one followed the chi-squared distribution and was not biased due to small *n* (Alexeyenko et al. [23] and unpublished results). In addition, this procedure was fast since multiple rounds of network randomization were not required – as compared to the algorithm earlier proposed by Maslov and Sneppen [24] and later implemented by us [13]. Of note, this simplification was only applicable when quantifying direct links *n*_*AGS-FGS*_. Evaluating any other, more complicated network pattern would warrant network randomization. The values were transformed to p-values and then adjusted for multiple testing [18]. We considered relations between AGS and FGS as significant when FDR <0.1, supported by at least 5 links.

We performed NEA with our software NEA.pl which was described in Merid et al. [15] and is available at http://research.scilifelab.se/andrej_alexeyenko/downloads.html. As a complement to the procedures described above, it performed binomial Gene Set Enrichment Analysis (GSEA) between the same gene sets, and reported the number of shared member genes.

In addition to group-wise AGS-FGS relations, NEA could analyze relations in a mode referred to as “*single gene vs. AGS”*, i.e. when a single gene was taken as if it were a pathway. Thus, the FGS was presented with a single gene, while the calculation otherwise was identical to [2]. This more specific mode facilitated the identification of both known pathway members and genes not assigned to any pathways. Of note, this is not possible in Gene Set Enrichment Analysis (GSEA).

### Simultaneous analysis of binding site enrichment and correlation of gene expression

We used the database of high-confidence predicted and experimental transcriptional factors (TF) and their targets HTRIdb [22] to analyze potential transcriptional regulators of the DE lists. We identified TFs most significantly linked to DE lists with our NEA software. In order to validate relevance of HTRIdb in this context, we looked at individual correlations in our expression datasets between profiles of identified TFs and respective DEGs across the 16 samples. After adjustment for expression profile length and multiple testing, we saw about 9-fold excess of correlated (FDR < 0.05) TF-target pairs compared to the pattern expected by chance (Additional file 1: Figure S5). Of note, here “by chance” implies presence of some non-zero fraction of correlated gene pairs, since many TFs would have global, unspecific effects. Hence taking into account existence of this truly regulation in certain random pairs, the formal level of FDR was close the empirical estimate.

## Results

### General design of the experiment

The fibroblasts were co-cultured with PC-3 cells. Their effect was quantified by scoring the number of cancer cells in each Petri dish after 72 h. The transcriptional consequences were evaluated in eight fibroblast lines including two sub-clones of the immortalized BjhTERT line Wh1 and Cr9 that differed in their capacity to inhibit cancer cells and six *ex vivo* fibroblast cell cultures that were sampled from either tumors or healthy tissues. The fibroblasts were harvested for mRNA expression analysis with and without PC-3 co-culture. All fibroblasts were confronted with tumor cells under the same conditions, and the experimental design included two points for each of the eight samples. Differential expression was detected for each gene on the chip in a number of statistical tests, namely by contrasting 1) the pre- versus post-confrontation transcriptomes and 2) by taking into account differences in inhibitory capacity of the different fibroblast samples. In addition, we were able to compare the *in vitro* and the *ex vivo* cell lines to determine their relevance as models of confrontation and inhibition.

**Fig. 1:**
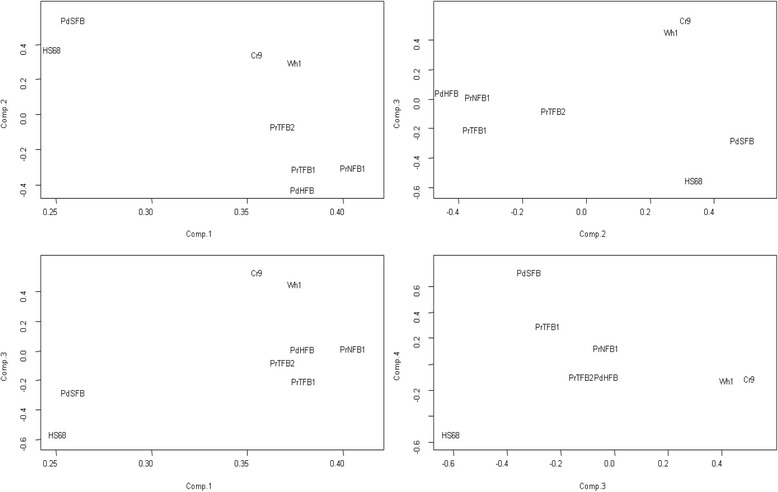
Overview of the transcriptional change during the confrontation with tumor cells in the eight fibroblast samples. The principal component (PC) analysis was performed of log_2_-transformed fold change expression values from Affymetrix for each gene in each of the 16 samples The text labels refer to cell sample IDs (see Methods) and are centered at the respective coordinates without offset. PCs from 1 to 4 are plotted as X and Y axes pair-wise. These four components took into account 49.9, 19.2, 11.2, and 7.4 % of the fold change variance, respectively. The analysis was meant to identify if any samples stand out compared to others in terms of overall expression

For an overview of the transcriptional changes during the confrontation of fibroblasts with PC-3 cancer cells, we performed the principal component analysis (PCA) of the fold change values on all genes in each of the eight samples. Fig. 1 shows that the samples distributed relatively evenly in the space of the first four principal components. Together, the four principal components took into account more than 87 % of total fold change variance. It is important to note that this analysis gave the most general view of the transcriptional landscape, where the most influential genes were those most different across all the 16 samples. In the following analysis, we will focus on more specific features, related to the confrontation with tumor cells and the capacity to inhibit the latter. Due to the common origin, the two *in vitro* samples Wh1 and Cr9 occupied close positions in each of the four PCA dimensions. However with the exception of component 3, they do not appear as an extreme group compared to the six *ex vivo* samples. Furthermore, Fig. 1 addresses the fact that some of the post-confrontation samples (namely those with Wh1, Cr9, and PdSFB) might contain relatively high amounts of tumor cells, and thus introduced a potential bias compared to the non-contaminated cells. However when compared to the rest of the samples, they did not stand out in any of the plotted components. From this we concluded that the cancer cell contamination was unlikely to have a larger impact on the analysis compared to other factors that determined variability between the samples.

### Confrontation with tumor cells

The confrontation of fibroblasts with PC-3 cells continuously for 72 h had a profound influence on the transcriptomes of the fibroblast lines, irrespectively of their inhibitory capacity. This warranted detailed functional investigation of differentially expressed genes (DEGs). The comparison of confronted vs. non-confronted fibroblasts across all the eight cell lines yielded an extensive list of 867 DEGs with a false discovery rate (q-value) < 0.05.

Applying the network enrichment analysis (NEA), we tested three DEG lists of different stringency: top 30, top 100, and top 300 genes that were most affected by the 72-h confrontation (as indicated above, the full list of significantly changing DEGs was much longer). Since the three lists gave comparable results that differed only by statistical power to detect pathways of known relevance, in the following we only report the analysis on the top 300 DEGs.

**Fig. 2:**
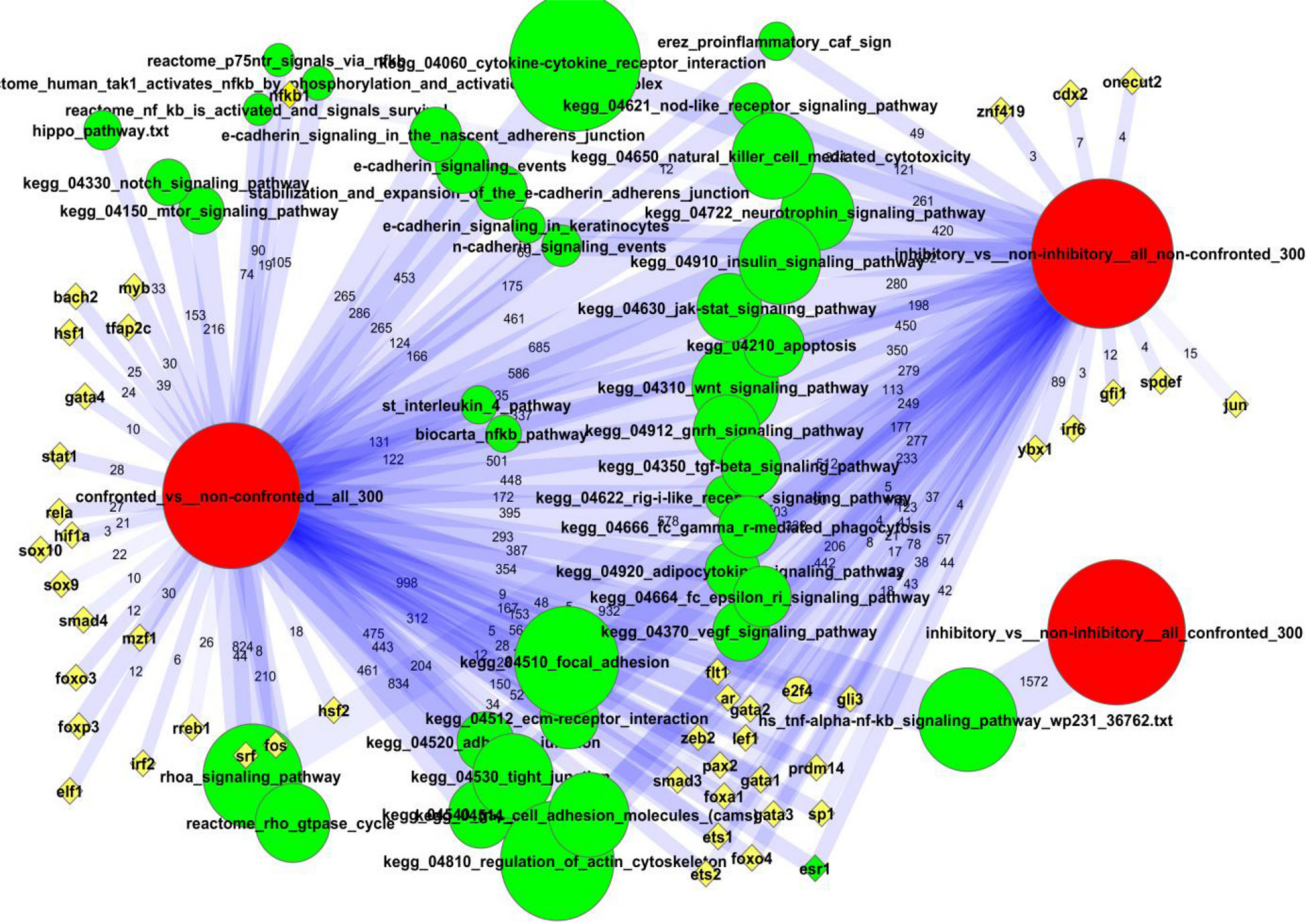
Network enrichment of experimentally perturbed genes. The summary of gene set (DEG vs. pathways or TFs) connections in the global network was performed using NEA. For description of the computational procedure see “Network enrichment analysis” in Methods. Lists of DE genes characterized either 1) expression change in the course of confrontation with tumor cells or 2) differential inhibition capacity of fibroblasts in regard of tumor cells. Red: lists of differentially expressed genes (DEG) from our experiment. Green: annotated pathways from public resources enriched in connections to DEGs. Yellow: individual transcription factors (TF) selected for their enrichment in network connections to DEGs. Lines are NEA-based summaries of individual, gene-gene network edges that connect DEGs and pathways between which significant network enrichment was detected (minimal NEA FDR < 0.01 and number of individual gene-gene links (see edge labels) at least 10 per pathway and at least 3 per TF). The image was created using software Cytoscape [39]

Multiple signaling pathways were enriched in network links to the DEGs, such as WNT, JAK-STAT, VEGF, apoptosis, insulin signaling, and others (Fig. 2). This was an important but not exclusive feature of the confrontation. Nearly all the same pathways were also identified in relation to inhibitory capacity (see next section). In the confrontation experiment, many pathways could share influential genes that contributed to enrichment, and the pathway scores could thereby positively correlate to each other. For example, NFKB1 was a member of three Reactome pathways related to NF-kB and each of the latter were enriched in our analysis (Additional file 2: File S1). Their member genes produced altogether 105, 90, and 74 individual links to DEGs per pathway (NEA FDR < 10^−7^ in each case). However, NFKB1 itself would be responsible for regulating not more than 19 out of 300 DEGs. To reveal both core pathway members like NFKB1 and other potential genes that might not have been assigned to any pathway, we retrieved network nodes that were most significantly linked to DEGs in the “single gene vs. DEG list” mode of NEA (see Methods). Using this approach, we identified a number of transcription factors (FOXO1, FOXO2, FOXO3, FOXO4, FOXP3, GATA1, GATA2, GATA4, SOX9, HSF2, SMAD3) and other potential regulators of the confrontational response (Table 1).

**Table 1:** Genes with potentially broad impact on the confrontation and the inhibitory capacity

Gene	Method of prioritization	Role	Description
*NFKBIA*	Confrontation DEG	Co-regulated by RELA	nuclear factor of kappa light polypeptide gene enhancer in B-cells inhibitor, alpha [Source: HGNC Symbol; Acc:7797]
*IL1B*	Confrontation DEG	Co-regulated by RELA	interleukin 1, beta [Source: HGNC Symbol; Acc:5992]
*RELT*	Confrontation DEG	Co-regulated by RELA	RELT tumor necrosis factor receptor [Source: HGNC Symbol; Acc:13764]
*BHLHE40*	Confrontation DEG	Co-regulated by RELA	basic helix-loop-helix family, member e40 [Source: HGNC Symbol; Acc:1046]
*FOS*	Connected to confrontation DEGs	Transcription factor	FBJ murine osteosarcoma viral oncogene homolog [Source: HGNC Symbol; Acc:3796]
*SRF*	Connected to confrontation DEGs	Transcription factor	serum response factor (c-fos serum response element-binding transcription factor) [Source: HGNC Symbol; Acc:11291]
*FOSB*	Confrontation DEG	Co-regulated by RELA	FBJ murine osteosarcoma viral oncogene homolog B [Source: HGNC Symbol; Acc:3797]
*CYR61*	Connected to confrontation DEGs	Pro-inflammatory signature (Erez et al., 2012)	cysteine-rich, angiogenic inducer, 61 [Source: HGNC Symbol; Acc:2654]
*IKBKB*	Connected to confrontation DEGs	Pro-inflammatory signature (Erez et al., 2012)	inhibitor of kappa light polypeptide gene enhancer in B-cells, kinase beta [Source: HGNC Symbol; Acc:5960]
*IKBKE*	Connected to confrontation DEGs	Pro-inflammatory signature (Erez et al., 2012)	inhibitor of kappa light polypeptide gene enhancer in B-cells, kinase epsilon [Source: HGNC Symbol; Acc:14552]
*IKBKG*	Connected to confrontation DEGs	Pro-inflammatory signature (Erez et al., 2012)	inhibitor of kappa light polypeptide gene enhancer in B-cells, kinase gamma [Source: HGNC Symbol; Acc:5961]
*IKBKG*	Connected to confrontation DEGs	Pro-inflammatory signature (Erez et al., 2012)	Inhibitor of kappa light polypeptide gene enhancer in B-cells, kinase gamma, isoform CRA_b [Source: UniProtKB/TrEMBL; Acc:D3DWY0]
*NR4A2*	Connected to confrontation DEGs	Pro-inflammatory signature (Erez et al., 2012)	nuclear receptor subfamily 4, group A, member 2 [Source: HGNC Symbol; Acc:7981]
*PDGFRA*	Connected to confrontation DEGs	Pro-inflammatory signature (Erez et al., 2012)	platelet-derived growth factor receptor, alpha polypeptide [Source: HGNC Symbol; Acc:8803]
*ANXA4*	inhibition DEG	Co-regulated by FOXA1	annexin A4 [Source: HGNC Symbol; Acc:542]
*CDK6*	inhibition DEG	Co-regulated by FOXA1	cyclin-dependent kinase 6 [Source: HGNC Symbol; Acc:1777]
*KAZN*	inhibition DEG	Co-regulated by FOXA1	kazrin, periplakin interacting protein [Source: HGNC Symbol; Acc:29173]
*RMND5A*	inhibition DEG	Co-regulated by FOXA1	required for meiotic nuclear division 5 homolog A (*S. cerevisiae*) [Source: HGNC Symbol; Acc:25850]
*TNS3*	inhibition DEG	Co-regulated by FOXA1	tensin 3 [Source: HGNC Symbol; Acc:21616]
*IL6*	Connected to inhibition DEGs	Potential regulator	interleukin 6 (interferon, beta 2) [Source: HGNC Symbol; Acc:6018]
*MAPK8*	Connected to inhibition DEGs	Potential regulator	mitogen-activated protein kinase 8 [Source: HGNC Symbol; Acc:6881]
*MAP2K4*	Connected to inhibition DEGs	Potential regulator	mitogen-activated protein kinase kinase 4 [Source: HGNC Symbol; Acc:6844]
*PRKCA*	Connected to inhibition DEGs	Potential regulator	protein kinase C, alpha [Source: HGNC Symbol; Acc:9393]
*YBX1*	Connected to inhibition DEGs	Transcription factor	Y box binding protein 1 [Source: HGNC Symbol; Acc:8014]
*GFI1*	Connected to inhibition DEGs	Transcription factor	growth factor independent 1 transcription repressor [Source: HGNC Symbol; Acc:4237]
*ESR1*	Connected to inhibition DEGs	Transcription factor	estrogen receptor 1 [Source: HGNC Symbol; Acc:3467]
*ETS1*	Connected to inhibition DEGs	Transcription factor	v-ets avian erythroblastosis virus E26 oncogene homolog 1 [Source: HGNC Symbol; Acc:3488]
*ETS2*	Connected to inhibition DEGs	Transcription factor	v-ets avian erythroblastosis virus E26 oncogene homolog 2 [Source: HGNC Symbol; Acc:3489]
*AR*	Connected to inhibition DEGs	Transcription factor	androgen receptor [Source: HGNC Symbol; Acc:644]
*GATA1*	Connected to inhibition DEGs	Transcription fact or	GATA binding protein 1 (globin transcription factor 1) [Source: HGNC Symbol; Acc:4170]
*GATA2*	Connected to inhibition DEGs	Transcription factor	GATA binding protein 2 [Source: HGNC Symbol; Acc:4171]
*GATA3*	Connected to inhibition DEGs	Transcription factor	GATA binding protein 3 [Source: HGNC Symbol; Acc:4172]
*PAX2*	Connected to inhibition DEGs	Transcription factor	paired box 2 [Source: HGNC Symbol; Acc:8616]
*JUN*	Connected to inhibition DEGs	Transcription factor	jun proto-oncogene [Source: HGNC Symbol; Acc:6204]
*STAT3*	Connected to inhibition DEGs	Transcription factor	signal transducer and activator of transcription 3 (acute-phase response factor) [Source: HGNC Symbol; Acc:11364]
*STAT5A*	Connected to inhibition DEGs	Transcription factor	signal transducer and activator of transcription 5A [Source: HGNC Symbol; Acc:11366]
*CAMK2A*	Confrontation DEG	Involved in TGFbeta signaling, associated with survival	calcium/calmodulin-dependent protein kinase II alpha [Source: HGNC Symbol; Acc:1460]
*CDK6*	Confrontation DEG	Involved in TGFbeta signaling, associated with survival	cyclin-dependent kinase 6 [Source: HGNC Symbol; Acc:1777]
*CDKN2B*	Confrontation DEG	Involved in TGFbeta signaling, associated with survival	cyclin-dependent kinase inhibitor 2B (p15, inhibits CDK4) [Source: HGNC Symbol; Acc:1788]
*NR4A1*	Confrontation DEG	Involved in TGFbeta signiling, associated with survival	nuclear receptor subfamily 4, group A, member 1 [Source: HGNC Symbol; Acc:7980]
*SMAD3*	Confrontation DEG	Involved in TGFbeta signiling, associated with survival	SMAD family member 3 [Source: HGNC Symbol; Acc:6769]
*TGFB1*	Confrontation DEG	Involved in TGFbeta signiling, associated with survival	transforming growth factor, beta 1 [Source: HGNC Symbol; Acc:11766]
*TGFB3*	Confrontation DEG	Involved in TGFbeta signiling, associated with survival	transforming growth factor, beta 3 [Source: HGNC Symbol; Acc:11769]
*EFNB2*	inhibition DEG	Associated with survival	ephrin-B2 [Source: HGNC Symbol; Acc:3227]
*TMEM220*	inhibition DEG	Associated with survival	transmembrane protein 220 [Source: HGNC Symbol; Acc:33757]
*TFAP2C*	inhibition DEG	Associated with survival	transcription factor AP-2 gamma (activating enhancer binding protein 2 gamma) [Source: HGNC Symbol; Acc:11744]
*RPSAP52*	inhibition DEG	Associated with survival	ribosomal protein SA pseudogene 52 [Source: HGNC Symbol; Acc:35752]
*SLC40A1*	inhibition DEG	Associated with survival	solute carrier family 40 (iron-regulated transporter), member 1 [Source: HGNC Symbol; Acc:10909]
*CYP1B1*	inhibition DEG	Associated with survival	cytochrome P450, family 1, subfamily B, polypeptide 1 [Source: HGNC Symbol; Acc:2597]
*CHAC1*	inhibition DEG	Associated with survival	ChaC, cation transport regulator homolog 1 (E. coli) [Source: HGNC Symbol; Acc:28680]
*CCL2*	inhibition DEG	Associated with survival	chemokine (C-C motif) ligand 2 [Source: HGNC Symbol; Acc:10618]
*PSAT1*	inhibition DEG	Associated with survival	phosphoserine aminotransferase 1 [Source: HGNC Symbol; Acc:19129]
*RCAN2*	inhibition DEG	Associated with survival	regulator of calcineurin 2 [Source: HGNC Symbol; Acc:3041]
*SCG2*	inhibition DEG	Associated with survival	secretogranin II [Source: HGNC Symbol; Acc:10575]
*AEBP1*	inhibition DEG	Associated with survival	AE binding protein 1 [Source: HGNC Symbol; Acc:303]
*MXRA8*	inhibition DEG	Associated with survival	matrix-remodelling associated 8 [Source: HGNC Symbol; Acc:7542]
*TBC1D1*	inhibition DEG	Associated with survival	TBC1 (tre-2/USP6, BUB2, cdc16) domain family, member 1 [Source: HGNC Symbol; Acc:11578]
*ITGA6*	inhibition DEG	Associated with survival	integrin, alpha 6 [Source: HGNC Symbol; Acc:6142]
*PLAUR*	inhibition DEG	Associated with survival	plasminogen activator, urokinase receptor [Source: HGNC Symbol; Acc:9053]

**Fig. 3:**
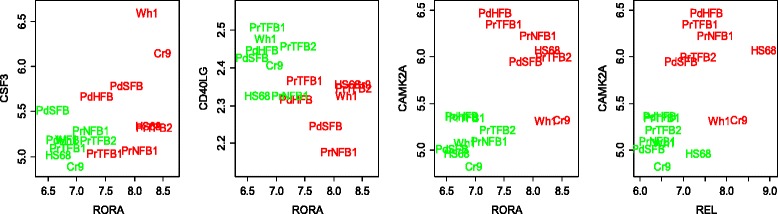
Expression patterns of transcription factors and their likely targets as related to confrontation with tumor cells. The plots are using log_2_-transformed expression values from Affymetrix. The text labels refer to cell sample IDs (see Methods) and are centered at the respective coordinates without offset. Green: gene expression before confrontation with tumor cells; Red: gene expression after 72 h of confrontation

Four cadherin-related pathways available in our collection were significantly associated with the confrontational response (NEA FDR < 10^−19^ for each of the pathways). In particular, we found that CAMK2A (calcium/calmodulin-dependent protein kinase II delta) was likely to be controlled by two transcription factors. The promoter region for CAMK2A contained binding sites for the transcription factors RORA and REL. RORA and REL were differentially expressed during the confrontation and their expression patterns were significantly correlated during that process (Pearson *r* = 0.777, *p*_*0*_ = 0.00018; Fig. 3) across the samples. Importantly, our network enrichment analysis was able to summarize bi-directional relations between DEGs and pathways. For example, we established, in a similar manner as above, that RORA could regulate two members of the KEGG pathway “Cytokine-cytokine receptor interaction”: colony stimulating factor CSF3 and CD40LG (the CD40 ligand). The latter genes were not included in the DEG list, because of lower significance of differential expression, but their expression pattern was still highly correlated with RORA expression.

We also found specific involvement of Rho signaling in the confrontational change. The transcription factor RELA, a co-factor of NFKB1, was likely to regulate a number of genes involved in Rho signaling. Based on the simultaneous analysis of binding sites and gene expression, one member of Rho signaling, the neuroepithelial cell transforming factor, NET1, appeared the most likely target of RELA (Additional file 1: Figure S1). Based on the same analysis, we concluded that RELA could also regulate the NF-kB inhibitor, NFKBIA, the interleukin IL1B, the TNF receptor RELT, and BHLHE40, which was one of the 12 CAF markers identified *in silico* [7]. Otherwise, regulation through Rho signaling could be implemented via the transcription factors (TF) FOS and SRF, which had predicted binding sites in 8 and 44 DEGs, respectively (NEA FDR <0.01 for each relation). However, Rho-signaling is not limited to these two TFs. The whole enrichment pattern of DEGs towards these pathways involved tens of genes and hundreds of functional links (Additional file 2: File S1).

We noticed that while binding sites for a number of transcription factors were enriched against the DEG list as a whole on the one hand, a subset of these factors, such as AR, MYB, ETS1, EGR1, GATA2, RELA and RREB1 may all target the same gene FOSB (FBJ murine osteosarcoma viral oncogene homolog) on the other hand. In particular, binding sites for RELA were identified in the FOSB promoter, while expression patterns of these two genes correlated significantly (Additional file 1: Figure S1).

**Fig. 4:**
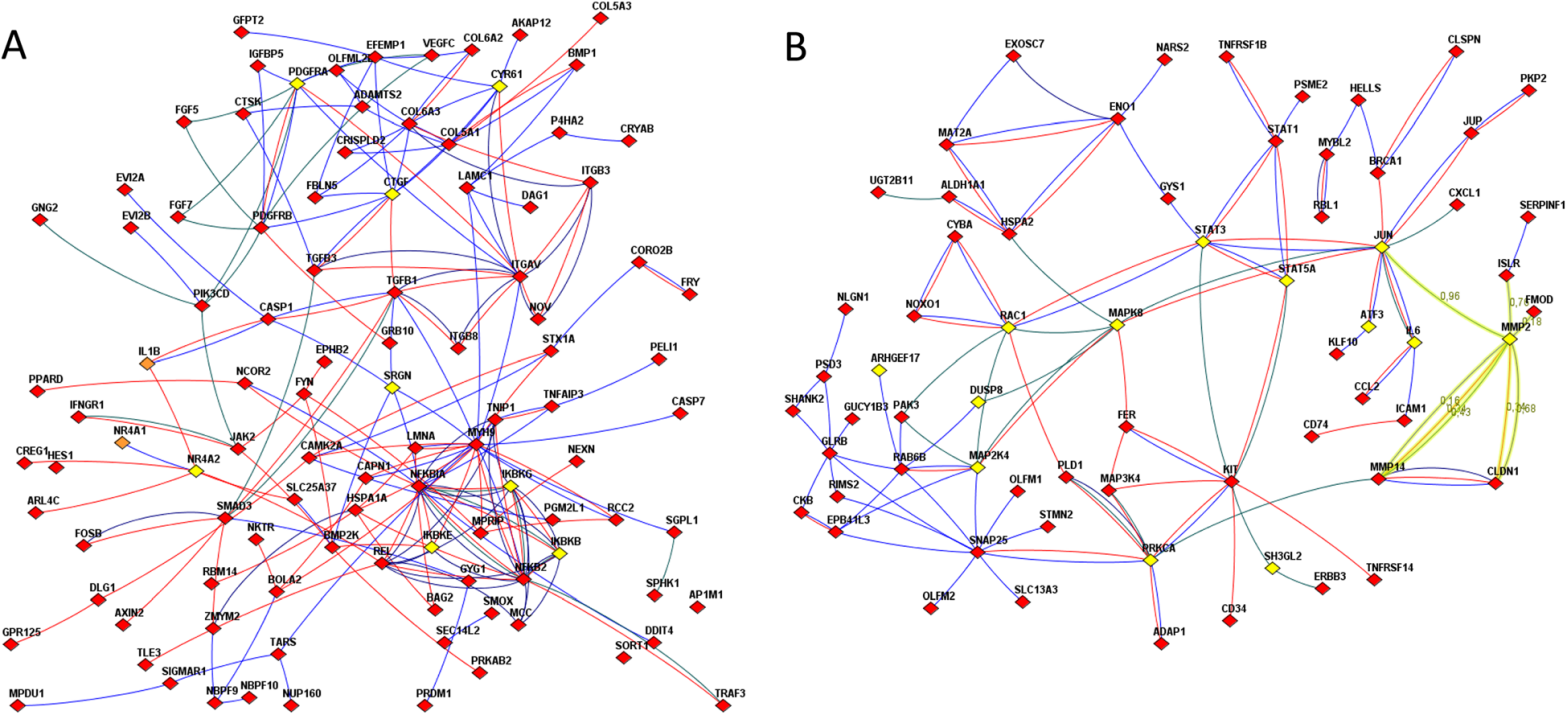
Functional relations of differentially expressed genes with each other and with members of important pathways, as displayed by FunCoup online resource [14]. **a** Genes differentially expressed under confrontation with tumor cells and pathway members. Nodes: Yellow, genes of the proinflammatory signature [27], and/or YAP/TAZ cascade [25], and/or Hippo pathway [26]. Red, confrontation-specific DEGs. Orange: genes that belong to both “yellow” and “red” categories Line color denotes origin of FunCoup evidence for the network edges (see also “Global interaction network” in Methods): Red, protein-protein interactions. Blue: co-expression of mRNA. Deep green: links in a KEGG pathway. Multiple lines between the same two nodes indicate that multiple lines of evidence and were treated as single edges in NEA. Lines with FunCoup confidence <0.1 for the individual component are not shown. **b** Genes differently expressed between 4 most and 4 least inhibitory fibroblast cultures before confrontation and pathway members. Yellow: the RhoA signaling pathway by PID database [30] and pathway for RhoD regulation of cytosceletal dynamics via WHAMM [31]. Red, DEGs between inhibitory and non-inhibitory fibroblast samples. The the line color, see the legend in A

Aragona et al. [25] pointed to a mechanical checkpoint of multicellular growth that acts via two transcriptional co-activators: the yes-associated protein YAP1 and tafazzin TAZ. The checkpoint mechanism continues downstream towards the Hippo pathway [26]. Indeed, we found (Fig. 4a) that two genes of the YAP/TAZ cascade (cysteine-rich angiogenic inducer 61, CYR61 and connective tissue growth factor, CTGF, a.k.a. hypertrophic chondrocyte-specific protein 24) were intertwined in the network of the Hippo pathway-related genes, MOB3C and TEAD3, and were also connected with proteins of the pro-inflammatory signature [27] interleukin IL1B, kinases IKBKB, IKBKE, IKBKG, nuclear receptors NR4A2 and NR4A2, PDGF receptor PDGFRA, and serglycin SRGN, as well as with tens of genes of the confrontation-specific DEG list including NFKB1, REL, and CASP1 (labeled red on Fig. 4a). This sub-network also displayed the involvement of TGF-beta signaling. Recently, Busch et al. [28] found that expression of TGFBR2 in CAFs of breast carcinoma could be associated with overall survival. In our analysis using The Cancer Genome Atlas data (reported in details below), TGFBR2 was associated with survival in ovarian and renal carcinoma. In addition, we identified associations between survival and expression of CDKN2B, BMP2, 5, 6, and 8A, as well as SMAD4, 6, 7, and 9. In Fig. 3A, one can see functional connections known from the literature (i.e. links present in the global network) for several members of the TGF-beta pathway that changed their expression upon confrontation, such as CAMK2A, CDK6, CDKN2B, NR4A1, SMAD3, TGFB1, and TGFB3.

### Transcriptional differences associated with tumor cell inhibition

We measured the degree of PC-3 cell inhibition after 72 h of in vitro co-culture with the fibroblasts. The fraction of surviving cells in the pure, non-confronted PC-3 culture was around 42 %, while in the fibroblast-confronted variants it ranged from 37.5 % (PdHFB) down to less than 20 % (PrNFB1). The Additional file 1: Figure S2 shows the averages and 95 % confidence intervals over the replicated measurements. All the pair-wise differences that we discuss and use below were significant at *p*-value <0.0001 by the Tukey post-hoc test of the one-way ANOVA [29].

#### Comparative analysis of in vitro and ex vivo fibroblasts

First we asked to what extent did the results obtained with *in vitro* and *ex vivo* fibroblasts agree with each other? The former, being immortalized lines, were more homogenous and stable. However, it had to be shown that they give results comparable to the *ex vivo* material.

We chose the low-inhibitory *in vitro* line Cr9 and its *ex vivo* low-inhibitory counterpart PrTFB2 and compared them to the highly inhibitory *in vitro* line Wh1 and its *ex vivo* counterpart PrNFB1. By plotting expression fold change values between the low versus high inhibitory cell lines before confrontation, we could see a certain similarity between *in vitro* and *ex vivo* cells: the positive correlation was weak (Spearman rank *r* = 0.105). After confrontation even this correlation vanished (*r* = −0.082) (Additional file 1: Figure S3).

**Fig. 5:**
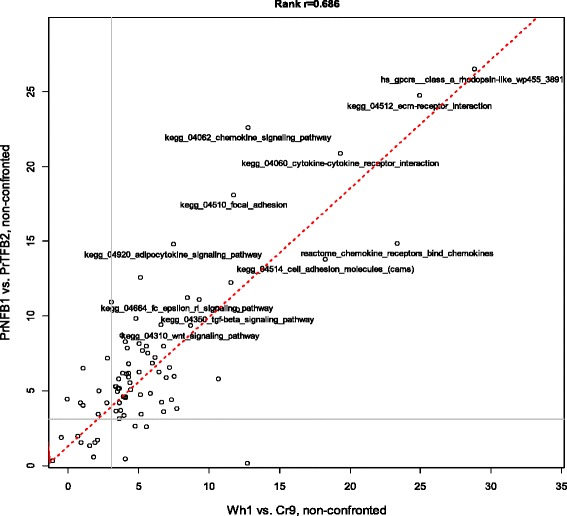
Pathway scores from network enrichment analysis for DEG lists compiled of genes most differing by mRNA expression level between low and high inhibitory cell lines *in vitro* (Wh1 vs. Cr9) and *ex vivo* (PrNFB1 vs. PrTFB2). The network analysis (see “Network enrichment analysis” in Methods) was performed on 300 genes with highest fold change between each pair of cell lines. Horizontal and vertical grey lines denote levels of significance in NEA as false discovery rate = 0.01. Red line displays the linear fit. The text labels are given for pathways with NEA FDR < 0.001 in the both conditions

Similarly to the principal component analysis presented above, these individual gene values were thus only weakly informative on the biological process. We therefore decided to investigate the DEGs using NEA. First, we had to demonstrate that the lists of DEGs between low and high inhibitory cells collected functionally relevant genes despite the absence of replicates. Assuming that the fold change values reported true differential expression, we expected to find functional interrelations between at least a fraction of the members of DEG lists. This could be proven with NEA by estimating internal connectivity. Indeed, we found that the respective DEGs were significantly interconnected with network links (NEA FDR <10^−8^ in all cases). As a negative control, NEA calculated enrichment for randomly generated gene sets of the same size and matching network topological properties. Expectedly, no enrichment was found for such sets. For final evidence that the expression differences between the low and high inhibitory cells in the *in vivo* and *ex vivo* pairs were consistent, we compared network enrichment of DEGs against relevant pathways. Each such pathway could potentially summarize a group of related DEGs, so that individual pathway-linked genes could be regarded as replicates in a statistical analysis with pathways as factor levels. Unlike the raw gene expression values, the pathway scores (Fig. 5) were indeed correlated: DEGs from *in vitro* and *ex vivo* fibroblasts were highly associated with the same pathways (Spearman rank R = 0.686, *p*_*0*_ < 10^−18^).

Here again, correlation was only found in the pre-confrontation transcriptomes, where it increased with the length of the DEG lists from 30 to 300, i.e. the statistical power grew with the sample size (Additional file 1: Figure S4a). In other words, gene ranking by fold change values between *in vitro* and *ex vivo* cells could differ, and the top 300 DEGs were a gene set sufficient to identify the involvement of all major pathways. The lack of correlation between confronted transcriptomes was specifically due to the wiping out of the differences in genes related to signaling in the *in vitro* cells after confrontation. Indeed, before confrontation multiple signaling pathways shaped the differences between the low and the high inhibitory cells. However after confrontation of the *in vitro* cells the list of enriched pathways (i.e. again those different between low and high inhibitory fibroblasts) only reflected basic maintenance processes, such as cell cycle, RNA-polymerase, protein export, ribosomal and proteasomal activities (not shown). Signaling pathways either received scores close to zero (no enrichment) or even became negative (depletion) (bottom plots at Additional file 1: Figure S4a). For comparison, nearly all pathways in the *ex vivo* lines remained equally enriched after confrontation (Additional file 1: Figure S4b). As a possible explanation of this difference, it may be recalled that the *in vitro* cells were derived from the same clone, whereas the *ex vivo* cells were sampled from different individuals and were not immortalized, and therefore prone to senescence, which could decrease their complexity.

#### Correlates of the inhibitory capacity

Compared to the confrontation experiment, the identification of genes associated with inhibitory capacity was more difficult. Against the background of dramatic changes upon the confrontation (the variance due to confrontation was much higher than that due to differential inhibitory capacity), it was statistically challenging to evaluate association of individual genes with just the inhibitory capacity. In order to find the inhibition-related features at a more general, pathway level, we produced DEG lists, each comprising 300 genes. Similarly to the described in the previous section, sets of top 300 genes were sufficient to detect pathway enrichment. The lists were intended to best distinguished between high and low inhibitory fibroblasts by grouping:

- Four of the most inhibitory (*in vitro* Wh1, and *ex vivo* PdSFB, PrNFB1, HS68) vs. four of the least inhibitory (*in vitro* Cr9, and *ex vivo* PdHFB, PrTFB1, PrTFB2) fibroblast cultures before confrontation and
- the same cultures after confrontation.

While reviewing the NEA results for the list of DEGs before confrontation (Fig. 2), we noticed that it was significantly linked to a range of pathways associated with cytokine/chemokine functionality and cell-cell interactions (focal adhesion, adherens junction, gap junction) and to general signaling processes, such as WNT, apoptosis, insulin signaling, JAK-STAT signaling and multiple other pathways found in the central area of Fig. 1. A more specific feature was that the RhoA signaling pathway as presented in the Pathway Interaction Database [30] and the pathway for RhoD regulation of cytoskeletal dynamics via WHAMM [31] were both significantly enriched in network links with the DEG list. The DEGs most involved in the connection pattern were interleukin IL6, kinases MAPK8, MAP2K4, and PRKCA, as well as transcription factors JUN, STAT3, and STAT5A. A number of transcription factors were associated with the DEG list by NEA, such as YBX1, GFI1, ESR1, ETS1, ETS2, AR, GATA1, GATA2, GATA3 and PAX2 (although expression of these genes was not altered significantly). Furthermore, by considering shared binding sites in promoters and correlation of gene expression, we established that FOXA1 might be a common regulator of a number of DEGs, such as annexin ANXA4, kinase CDK6, kazrin KAZN, the gene RMND5A, “required for meiotic nuclear division 5 homolog A (*S. cerevisiae*)”, and tensin TNS3 (Additional file 1: Figure S1). Most of the pathways overlapped with those that characterized the response to confrontation with tumor cells, although details of these two distinct modes of regulation differed at the level of individual genes.

### Association of the most prominent genes with survival

We used public transcriptomics and clinical data from The Cancer Genome Atlas (TCGA, 2010) with a two-fold goal:

1. To validate the relevance of our transcriptomics and network analyses, and
2. To further investigate potentially relevant properties of the identified genes and pathways.

The TCGA data were based on samples of primary solid tumors, with unknown fractions of fibroblasts, which could vary over a broad range. To what extent were the samples representative of the fibroblast transcriptomes and thus tumor cell inhibition that would naturally go on *in vivo*? On the one hand, we expected this to be the case since gene expression had been successfully correlated to overall survival in the breast carcinoma by Busch et al. [28] on CAF transcriptomes and by Frings et al. [32] on complex tumor samples. On the other hand, a study by Liu et al. [33] reported a direct association between the size of the stromal fraction and survival in early cervical carcinoma. We reasoned that in such analyses mRNA expression would be nearly always affected by extra molecular factors and spatial cell-to-cell interactions beyond our control. Therefore in absence of assumptions about specific biases and by combining analyses from as many as ten different cancer types, we hoped to reveal associations with the overall patient survival. If the setup was not appropriate, we could not expect more associated genes than was expected by chance in a set of randomly picked genes.

For the analysis, we were able to use ten different cancers and four high-throughput platforms (two microarray manufacturers and two RNA-sequencing procedures), which in total gave 24 cancer/platform combinations. For technical and biological reasons, these were differently informative on the overall survival. By visually reviewing genome-wide p-value distributions, we found that each dataset could report from few to thousands truly correlated genes (i.e. significant after adjustment for multiple testing). We found significant concordance of genes between the datasets. Each individual gene received a combined p-value from all these comparisons [34], which was then in its turn adjusted for multiple testing. This procedure allowed unbiased global (over the 10 cancer types) ranking for the majority of genes (more than 15,000 by requiring that expression was available from every TCGA set as well as from our microarray dataset).

We found that a number of DEGs, both from the confrontation- and inhibition-specific lists, were correlated with relapse-free survival in a number of solid tumors, such as renal and breast carcinoma, as well as squamous cell carcinomas of head and neck and lung. However, relevance of our DEG lists to survival could be proven only if they would be significantly overrepresented, compared to randomly selected genes. First, we identified enrichment of survival-associated genes for the “inhibitory vs. non-inhibitory” DEG lists (2.3-fold for combined p-values <10^−6^). The comparison involving only the selected pairs Wh1 vs. Cr9 and PrNFB1 vs. PrTFB2 yielded even more enrichment (four-fold). For comparison, the DEG list for the contrast “before vs. after confrontation” did not show any enrichment (although its network neighbors did, as explained below). This was a meaningful outcome, as our biological hypothesis assumed that an active role should belong to the inhibition*.*

Importantly, inhibition-related differences between the *in vitro* and *ex vivo* cells again shared similar expression features. While the lists from each comparison, i.e. DEGs from Wh1 vs. Cr9 and then separately DEGs from PrNFB1 vs. PrTFB2, shared 59 genes in total, 11 of the latter were found to be correlated with survival in TCGA (combined p-value <0.001), namely EFNB2, TMEM220, TFAP2C, RPSAP52, SLC40A1, CYP1B1, CHAC1, CCL2, PSAT1, RCAN2, and SCG2.

The analysis of the DEG list that combined the *in vitro* and *ex vivo* cells (Wh1 vs. Cr9 and PrNFB1 vs. PrTFB2) identified a number of genes with less obvious roles: AEBP1 (adipocyte enhancer-binding protein 1), TBC1D1, previously associated with obesity [35], MXRA8 (limitrin, matrix-remodelling associated 8), which might be involved in the formation of the blood-brain barrier [36], PLAUR (plasminogen activator, urokinase receptor), and integrin ITGA6.

We also considered association with survival for genes from a collection of 94 pathways that were relevant to signaling in general and to the formation and functioning of extra-cellular matrix, cell junctions and fibroblast activity according to current knowledge (Additional file 2: File S1). Most of these pathways were not associated with survival. Remarkably however, such pathways as “Rho GTPase cycle”, “E-cadherin signaling in the nascent adherens junction”, “Adherens junction”, as well as GO term “Rho GTPase activator activity”, were enriched in survival-associated genes from 2- to 4-fold. In addition, 3 out of our 12 *in silico* CAF markers [7], 12 genes of the pro-inflammatory CAF signature [27], 16 genes of the Hippo pathway [26], and 3 genes of the YAP/TAZ pathway [25] were found in the overrepresented sets. Next, we investigated if network neighbors of the members of our DEG lists were enriched among the survival-associated genes (Additional file 2: File S1). Such gene groups were often even more overrepresented than DEGs and pathway members by themselves, up to 7–8 folds. A similar network analysis identified network neighbors of the pathways mentioned above, which again increased the number and significance of the overlap. The full list of DEGs and pathway members associated survival can be found in the Additional file 2: File S1.

Based on our analysis we propose that the group of genes discovered via network enrichment against survival genes (both individually and as a whole group) should be subjected to further investigation by the combination of our methods (Table 1).

## Discussion

We confronted fibroblasts with cancer cells and interpreted transcriptomes of the former, before and after the confrontation, using statistical and systems biology procedures. Against the background of histological, clinical, and genetic differences between the fibroblasts, we gained insights into the biological processes in question using a self-controlled, differential experimental design.

The network enrichment analysis that we applied combined effects of both up- and down-regulated genes. This allowed summarizing features to the pathway level. When enrichment of a certain pathway was significant for different DEG lists (e.g. those that characterized confrontation and inhibition), individual DEGs behind the enrichments might still vary. In other words, the algorithm of NEA allowed identifying same pathways via different, condition-specific gene sets, which might partially overlap with each other. This increased the sensitivity of the analysis and the generality of the conclusions, but posed an extra challenge for the interpretation. Which individual genes should be picked up for experimental validation? Since our study was based on transcriptome analysis, we focused on potential regulators of transcription. We started from an exploratory analysis at the level of whole pathways, and demonstrated that individually insignificant genes can produce a network connectivity pattern that was extremely unlikely to occur in a random set of genes. For comparison, the traditional gene set enrichment analysis [21] was much less efficient on the same DEG lists, because only very few DEGs were direct members of respective pathways. Many relations between individual transcription factors and DEGs were further confirmed by gene expression analysis.

We found that many functional links between a TF and a downstream gene could be confirmed by gene expression correlation. However, even when such correlation could not be identified, functional relations were still likely, since an up-stream TF might be constitutively expressed and regulated, e.g. by physical protein interactions or co-binding with other TFs.

Cells from different original sources served as biological replicates in this experimental design. Despite the potential pitfalls of this setting, such as loss of statistical power (many false negatives) and spurious positive results due to excessive biological variability, we likely captured the general features that could be recurrently reproduced in future research. This was helped by our self-controlled paired analysis design where pre-confrontation and post-confrontation data of the same cell line were directly compared. Importantly, NEA was indispensable here as well, due to its ability to integrate results both at the individual pathway and signaling network levels. Using NEA, we could confirm the validity of DEG lists in two ways: 1) by detecting the internal coherence of the lists, i.e. significant enrichment of connections (network edges) within the list compared to a pattern expected in a random gene list, and 2) by observing multiple pathways significantly enriched in relations to the DEG genes compared to the number of similarly enriched pathways for a random gene list.

Our use of cell lines as biological replicates was similar to the approach of high-throughput profiling in large cancer projects. It is usually done on either unique primary tumor samples or cancer cell lines of very different origin (an example can be found in the analysis of primary tumors versus metastasis sites of ovarian tumor by Malek et al. [37]), which permits the evaluation of random effects of biological samples, i.e. biological variation in general rather than features of specific phenotypes. We note that the range of cells that could potentially interact with tumor ones is not even limited to fibroblasts and includes e.g. prostate adipocytes [38].

We also addressed the problem of contamination of post-confrontation fibroblasts with cancer cells and the potential bias in the transcriptome measurements in the confronted cultures. The overall signal was strong and reproducible both in the potentially contaminated and in the non-contaminated samples. Bias in the analysis of inhibition could be ruled out by common features between Wh1 versus Cr9 (both contaminated) on the one hand and PrNFB1 versus PrTFB2 (both uncontaminated) samples. In parallel, the principal component analysis (Fig. 1) did not indicate any specifically strong fraction of variability due to the presence of cancer cells. We reported only genes reproduced in the both comparisons.

The changes due to confrontation were much more drastic than those associated with differences in inhibitory capacity, which warranted different approaches to the analysis and the statistical validation. At the same time, the two types of analyses highlighted an overlap between the individual genes, signaling systems and processes, although with different functional implications. We detected association of our DEGs with cell-cell interaction (in the form of focal adhesion, adherens junction, and gap junction pathways) and signaling systems that should be involved in the confrontation process according to previous research, such as Rho signaling [7, 31] YAP/TAZ cascade [25], the proteins of the pro-inflammatory signature [27], and biomarkers of CAFness [7]. Among pathways and biological processes associated with differential inhibitory capacity were, again, those responsible for cell-cell interactions, the RhoD pathway [31], and the pro-inflammatory signature [27].

The set of genes that we discussed and prioritized in this work are summarized in Table 1. The general rule for inclusion into this table was the potential to have a broader impact on either the confrontational response or the inhibitory capacity and thus to allow reexamining, in future experiments, the effects observed in the present confrontation assay.

Using this gene list, we and others would be able to connect the global, pathway-level view on the interaction between fibroblasts and cancer cells with gene-focused approaches of experimental research and eventual development of novel anti-cancer drugs.

## Additional files

Additional file 1: Figure S1. Correlation patterns of transcription factors and their potential targets identified in the network analysis. **Figure S2.** Inhibition scores as fraction of survived PC-3 cells after 72 h co-cultivation with fibroblasts. The technique is described by Alkasalias et al. [8]. **Figure S3.** Comparison of differential expression using raw gene expression values between low and high inhibitory cell lines *in vitro* (fold change values Wh1 vs. Cr9) and *ex vivo* (fold change values PrNFB1 vs. PrTFB2). **Figure S4.** Pathway scores from network enrichment analysis of most altered genes between low and high inhibitory cell lines in vitro (Wh1 vs. Cr9) and ex vivo (PrNFB1 vs. PrTFB2). **Figure S5.** Using co-expression to validate transcriptional regulation reported by database HTRIdb. Additional file 2:
**Supplementary tables.**
