# Supplementary material for: Confrontation of fibroblasts with cancer cells in vitro: gene network analysis of transcriptome changes and differential capacity to inhibit tumor growth
Source: J Exp Clin Cancer Res. 2015 Jun 18;34(1):62. doi: 10.1186/s13046-015-0178-x (PMC4472614; doi:10.1186/s13046-015-0178-x)

**Supplementary figures**

Supplementary Figure 1. Correlation patterns of transcription factors and their potential targets identified in the network analysis.

X-axis: transcription factors; Y-axis: target genes.

Green: expression in the original fibroblast *in vitro* samples; Red: expression after confrontation with tumor cells.

Affymetrix log_2_(intensity) values are used for the plots.

Supplementary Figure 2. Inhibition scores as fraction of survived PC-3 cells after 72h co-cultivation with fibroblasts. The technique is described by Alkasalias et al. (2014).

Wh1

Cr9

PrNFB1

PrTFB2

PdSFB

PdHFB

HS68

PC3 alone

0.10

0.15

0.20

0.25

0.30

0.35

0.40

0.45

0.50

Supplementary Figure 3. Comparison of differential expression using raw gene expression values between low and high inhibitory cell lines *in vitro* (fold change values Wh1 vs. Cr9) and *ex vivo* (fold change values PrNFB1 vs. PrTFB2).


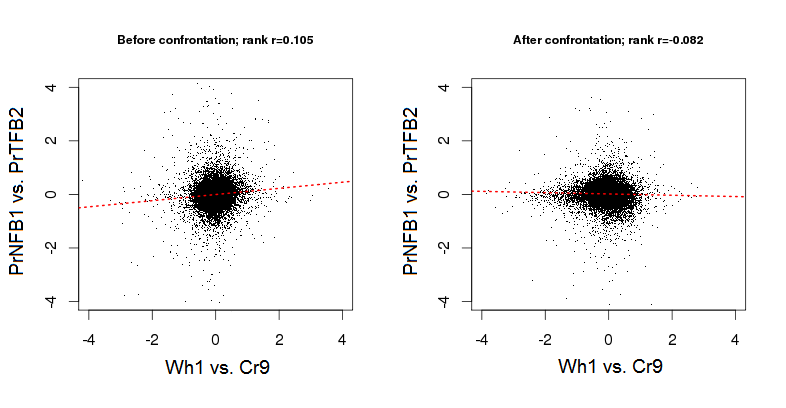


Supplementary Figure 4. Pathway scores from network enrichment analysis of most altered genes between low and high inhibitory cell lines in vitro (Wh1 vs. Cr9) and ex vivo (PrNFB1 vs. PrTFB2).

After ranking by fold change, top 30, 100, and 300 genes were derived for the network analysis.

A. Comparison of *ex vivo* and *in vitro* transcriptomes before and after confrontations in three DEG lists of different length.

B. Comparison of *ex vivo* transcriptomes before and after confrontations with the maximal (300) length gene of lists.

A


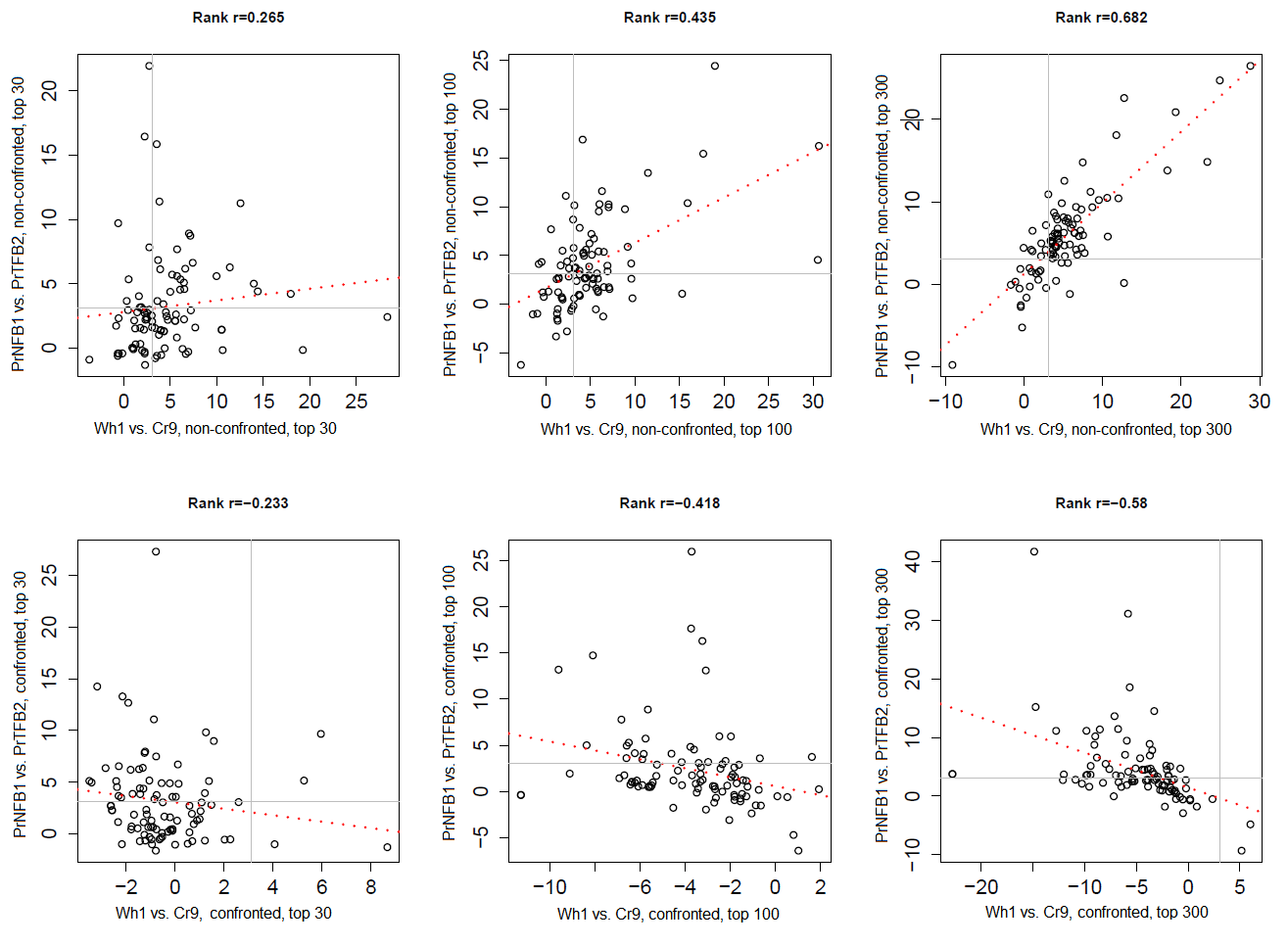


B

Supplementary Figure 5. Using co-expression to validate transcriptional regulation reported by database HTRIdb.

Upper pane: Distribution of estimates of significant co-expression (p-values adjusted by Benjamini and Hochberg, 1995) in pairs “transcription factor – target gene” from HTRIdb [15] within our microarray dataset.

Lower pane: Same distribution, but in randomly permuted pairs.


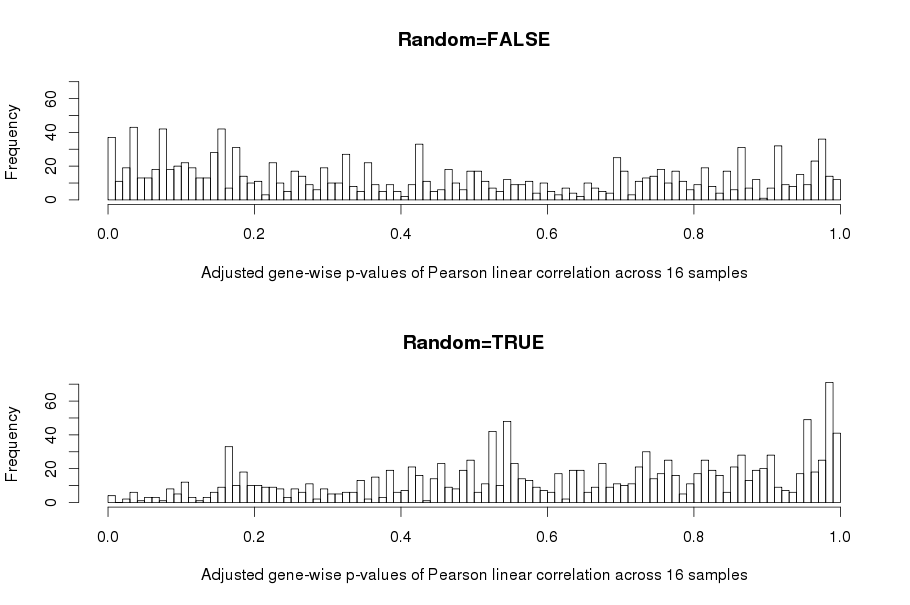

Supplement: Additional file 1: Figure S1. — Correlation patterns of transcription factors and their potential targets identified in the network analysis. Figure S2. Inhibition scores as fraction of survived PC-3 cells after 72 h co-cultivation with fibroblasts. The technique is described by Alkasalias et al. [8]. Figure S3. Comparison of differential expression using raw gene expression values between low and high inhibitory cell lines in vitro (fold change values Wh1 vs. Cr9) and ex vivo (fold change values PrNFB1 vs. PrTFB2). Figure S4. Pathway scores from network enrichment analysis of most altered genes between low and high inhibitory cell lines in vitro (Wh1 vs. Cr9) and ex vivo (PrNFB1 vs. PrTFB2). Figure S5. Using co-expression to validate transcriptional regulation reported by database HTRIdb. [file 13046_2015_178_MOESM1_ESM.docx]
